# Growing old with antiretroviral therapy or elderly people in antiretroviral therapy: two different profiles of comorbidity?

**DOI:** 10.1186/s12879-022-07739-y

**Published:** 2022-09-23

**Authors:** Paolo Maggi, Giuseppe Vittorio De Socio, Barbara Menzaghi, Chiara Molteni, Nicola Squillace, Lucia Taramasso, Marta Guastavigna, Giulia Gamboni, Giordano Madeddu, Francesca Vichi, Antonio Cascio, Eleonora Sarchi, Giovanni Pellicanò, Canio Vito Martinelli, Benedetto Maurizio Celesia, Laura Valsecchi, Roberto Gulminetti, Giovanni Cenderello, Andrea Parisini, Leonardo Calza, Katia Falasca, Giancarlo Orofino, Elena Ricci, Antonio Di Biagio, Paolo Bonfanti

**Affiliations:** 1 Department of Infectious Disease, University of Campania Luigi Vanvitelli, Naples, Italy; 2 Clinic of Infectious Diseases, Department of Medicine 2, Azienda Ospedaliera di Perugia, Santa Maria Hospital, Perugia, Italy; 3 Unit of Infectious Diseases, ASST della Valle Olona, Busto Arsizio Hospital, Busto Arsizio, Italy; 4 Infectious Disease Unit, Ospedale A. Manzoni, Lecco, Italy; 5 Infectious Diseases Clinic, San Gerardo Hospital, University of Milano-Bicocca, Monza, Italy; 6 Infectious Disease Clinic, IRCCS Policlinico San Martino Hospital, Genoa, Italy; 7 Unit of Infectious Diseases, “Divisione A”, Amedeo di Savoia Hospital, Turin, Italy; 8 Unit of Infectious and Tropical Diseases, Department of Medical, Surgical and Experimental Sciences, University of Sassari, Sassari, Italy; 9 Infectious Diseases Department, SOC 1, USLCENTRO Firenze, Santa Maria Annunziata Hospital, Florence, Italy; 10 Department of Health Promotion, Mother and Child Care, Internal Medicine and Medical Specialties, University of Palermo, Palermo, Italy; 11 Infectious Diseases Unit, SS. Antonio e Biagio e Cesare Arrigo Hospital, Alessandria, Italy; 12 Unit of Infectious Diseases, Department of Human Pathology of the Adult and the Developmental Age ‘G. Barresi’, University of Messina, Messina, Italy; 13 SOD Malattie Infettive e Tropicali AOU Careggi, Florence, Italy; 14 Unit of Infectious Diseases, University of Catania, ARNAS Garibaldi, Catania, Italy; 15 Infectious Disease Unit, ASST Fatebenefratelli Sacco, Milan, Italy; 16 Fondazione IRCCS Policlinico San Matteo, University of Pavia, Pavia, Italy; 17 Infectious Diseases Department, Sanremo Hospital, Sanremo, Italy; 18 Department of Infectious Diseases, Galliera Hospital, Genoa, Italy; 19 Department of Medical and Surgical Sciences, Clinics of Infectious Diseases, S. Orsola-Malpighi Hospital, “Alma Mater Studiorum” University of Bologna, Bologna, Italy; 20 Clinic of Infectious Diseases, Department of Medicine and Science of Aging, University ‘G. d’Annunzio’ Chieti-Pescara, Chieti, Italy; 21 Fondazione ASIA Onlus, via Garibaldi, 13, 20090 Buccinasco, MI Italy

**Keywords:** HIV, Multimorbidity, ART exposure, Age

## Abstract

**Background:**

In persons living with HIV (PLWH), the burden of non-communicable chronic diseases increased over time, because of aging associated with chronic inflammation, systemic immune activation, and long-term exposure to the combination antiretroviral therapy (ART).

**Methods:**

To explore the association of chronological age, age at first ART, and exposure to ART with non-communicable chronic diseases, we performed a cross-sectional analysis to evaluate the prevalence of comorbidities in patients enrolled in the SCOLTA Project, stratified by groups of chronological age (50–59 and 60–69 years) and by years of antiretroviral treatment (ART, ≤ 3 or > 3 years).

**Results:**

In 1394 subjects (23.8% women), mean age at enrollment was 57.4 (SD 6.5) years, and at first ART 45.3 (SD 10.7). Men were older than women both at enrollment (57.6 vs 56.8, p = 0.06) and at first ART (45.8 vs 43.6, p = 0.0009). ART duration was longer in women (13.1 vs 11.7 years, p = 0.01). The age- and sex-adjusted rate ratios (aRRs, and 95% confidence interval, CI) showed that longer ART exposure was associated with dyslipidemia (aRR 1.35, 95% CI 1.20–1.52), hypertension (aRR 1.52, 95% CI 1.22–1.89), liver disease (aRR 1.78, 95% CI 1.32–2.41), osteopenia/osteoporosis (aRR 2.88, 95% CI 1.65–5.03) and multimorbidity (aRR 1.36, 95% CI 1.21–1.54). These findings were confirmed in strata of age, adjusting for sex.

**Conclusions:**

Our data suggest that longer ART exposure was associated with increased risk of dyslipidemia, hypertension, and osteopenia/osteoporosis, hence the presence of multimorbidity, possibly due to the exposition to more toxic antiretrovirals. We observed different comorbidities, according to ART exposure and age.

**Supplementary Information:**

The online version contains supplementary material available at 10.1186/s12879-022-07739-y.

## Background

In recent years, the prevention and the early treatment of co-morbidities among persons living with HIV infection (PLWH) has become a major issue in the management of these patients. In fact, a number of non-communicable diseases (cardiovascular [1], renal [2], neurocognitive [3, 4], bone disease [5], non-AIDS related cancers [6]) are more frequently observed among PLWH with respect to the general population as a consequence of aging, higher exposure to behavioral factors (such as smoking, substance abuse and concurrent sexually transmitted diseases), chronic inflammation, systemic immune activation, and long-term exposure to the combination antiretroviral therapy (ART) [7–9]. Moreover, the presence of multiple chronic co-morbidities and co-medication [10], could determine further difficulties in their management.

Aimed at the purpose of defining the possible different profiles in terms of type and quantity of non-communicable diseases among PLWH, we described the distribution of comorbidities according to their chronological age, and the length of exposition to ART.

## Methods

This was a cross-sectional analysis of baseline data from the Surveillance COhort Long-Term Toxicity of Antiretrovirals/antivirals (SCOLTA) project, an ongoing prospective cohort study [11]. Briefly, subjects were enrolled if they were aged 18 years or more, in need of initiating a cohort drug and gave their informed consent to participate into the study, which was approved by the institutional ethics committee of the coordinating center in 2002 and amended in 2013 and 2019. At enrollment, information on type and duration of previous ART were collected, as well as on diagnosed comorbidities. All new patients in SCOLTA are systematically screened for comorbidities and, in the follow up, all non-communicable diseases of new insurgence are registered.

Included comorbidities (diagnosed with laboratory, clinical or instrumental data and/or drug tracing criteria) were diabetes mellitus; hypertension; previous cardiovascular diseases, defined as diagnosed atherosclerosis, carotid plaques, hypertensive cardiomyopathy, ischemic cardiopathy, atrial fibrillation, cardiac hypertrophy, tachycardia, and history of brain hemorrhage, acute myocardial infarction, transitory ischemic attack, or cardiac failure; dyslipidemia, based on serum cholesterol (> 200 mg/dL) and triglycerides (> 150 mg/dL) values; renal impairment (diagnosis of chronic kidney disease or estimated glomerular filtration (eGFR) < 60 mL/min, according to the Modification of Diet in Renal Disease formula); central nervous system (CNS) disturbance, based on medical history of anxiety, depression, psychosis; liver disease, such as any chronic hepatitis or detectable hepatitis C virus (HCV) RNA; osteopenia/osteoporosis, based on bone density scan and drug-tracing criteria; epilepsy, based on specialist’s diagnosis; lung disease (asthma, chronic obstructive pulmonary disease, emphysema) based on specialist’s diagnosis; hypovitaminosis D; history of non-AIDS defining cancers, based on medical history and oncologic follow up; obesity, defined as body mass index > 30.0 kg/m^2^. Multi-morbidity was defined as the presence of two or more co-morbidities other than HIV infection.

To focus on older subjects, we selected participants enrolled since 2013 and aged 50 or more years at study entry. To investigate the effect of ART exposure, we compared PLWH with low exposure (naïve or up to 3 years of treatment) and PLWH with moderate-high exposure (more than 3 years).

Continuous variables were described as means (and standard deviation, SD) if normally distributed, and as medians (and interquartile range, IQR) if not normally distributed. They were compared using parametric and non-parametric tests, respectively. Categorical variables were described as frequency (and percentage, %) and their distribution was compared using the Chi-square test (or Fisher exact test, as appropriate).

Using unconditional multiple logistic regression, we estimated the rate ratios (RRs) and the corresponding 95% confidence intervals (CIs) for diabetes, hypertension, other cardiovascular diseases (CVDs), dyslipidemia, chronic kidney disease (CKD), CNS disturbance, bone loss (osteopenia and osteoporosis), non-AIDS-related malignancies and multimorbidity in categories of ART duration (> 3 years vs ≤ 3 years). To account for potential confounders, we included terms for age, sex at birth (as factors related to most chronic comorbidities) and HCV coinfection (except for liver disease since HIV coinfection is classified as such). All the analyses were performed with the SAS software, version 9.4 (SAS Institute, Inc., Cary, NC, USA).

## Results

Out of 3040 patients enrolled since 2013, we excluded 1646 (54.1%) who were younger than 50 years at study entry. One thousand three hundred and ninety-four were eligible for this analysis, 984 (70.6%) aged 50–59 years and 410 (29.4%) aged 60 years or more.

Mean age was 57.4 (SD 6.5) years at study entry, and 45.3 (SD 10.7) years at first ART. Both were higher in men than in women (57.6 vs 56.8, p = 0.06, and 45.8 vs 43.6, p = 0.0009). On the contrary, ART duration was longer in women (13.1 vs 11.7 years, p = 0.01).

Baseline patients’ characteristics are reported in Table 1, by ART exposure. Groups were different in term of HCV positivity, risk factor for HIV acquisition, median CD4 + cell count (lower in low ART exposure), and mean eGFR (higher in low ART exposure). One hundred and sixty-eight (12.0%) patients did not present any comorbidities.

**Table 1:** Baseline characteristics of 1394 subjects aged ≥ 50 years enrolled in the SCOLTA Project between 2013 and 2021

Patients’ characteristics	ART exposure				
	≤ 3 years		> 3 years		p
	N	%	N	%	
Total	323	23.2	1071	76.8	–
Age, years (mean, SD)	57.1 ± 6.3		57.5 ± 6.6		0.24
Sex					
Male	256	79.3	806	75.3	0.14
Female	67	20.7	265	24.7	
Caucasian ethnicity	304	94.1	1033	96.4	0.06
BMI, Kg/m^2^ (mean, SD)	24.3 ± 3.7		24.9 ± 4.2		0.047
Risk factor for HIV acquisition					
IDU	41	12.7	334	31.2	< 0.0001
Heterosexual	140	43.3	377	35.2	
Homosexual	106	32.8	236	22.0	
Other/unknown	36	11.2	124	11.6	
HCV positive	44	14.6	388	37.6	< 0.0001
CDC Stage					
A	167	51.7	453	42.3	0.14
B	68	21.0	344	32.1	
C	88	27.4	274	25.6	
Detectable HIVRNA (exp)	23	13.4	140	13.1	0.91
CD4 + count, cell/mL (median, IQR)	399	211–642	634	442–868	< 0.0001
eGFR, mL/min (mean, SD)	89.3 ± 24.3		84.8 ± 22.6		0.002
Comorbidities					
0	76	23.5	92	8.6	< 0.0001
1	94	29.1	280	26.1	
≥ 2	153	47.4	699	65.3	

*ART* antiretroviral treatment, *eGFR* estimated glomerular filtration rate, *HCV* human C hepatitis virus, *IDU* intravenous drug use, *IQR* interquartile range, *SD* standard deviation

We found a significantly higher frequency of dyslipidemia, hypertension, hypovitaminosis D, chronic liver disease, osteopenia/osteoporosis, epilepsy and multimorbidity in PLWH with longer ART exposure (Table 2). After accounting for age class (50–59 and ≥ 60 years), sex and HCV coinfection (except for liver disease), all associations but epilepsy were confirmed.

**Table 2:** Comorbidities by ART exposure: prevalence and adjusted rate ratios (aRR) for morbidity risk (reference category ≤ 3 years)

Comorbidity	Total		ART exposure					> 3 vs ≤ 3 years	
			≤ 3 years		> 3 years		P	aRR	95% CI
	N	%	N	%	N	%			
	1394	100	323	23.2	1071	76.8			
Dyslipidemia	909	65.2	169	52.3	740	69.1	< 0.0001	1.35	1.20–1.52
Hypertension	440	31.6	73	22.6	367	34.3	< 0.0001	1.52	1.22–1.89
Hypovitaminosis D	342	24.5	46	14.2	296	17.6	< 0.0001	1.86	1.40–2.49
Liver disease	285	20.4	42	13.0	243	22.7	0.0002	1.78	1.32–2.41
CNS disturbance	201	14.4	52	16.1	149	13.9	0.33	0.78	0.56–1.05
Renal impairment	152	10.9	28	8.7	124	11.6	0.14	1.39	0.92–2.09
Osteopenia/osteoporosis	148	10.6	13	4.0	135	12.6	< 0.0001	2.88	1.65–5.03
Diabetes	124	8.9	29	9.0	95	8.9	0.95	1.06	0.70–1.60
Other CVD	114	8.2	19	5.9	95	8.9	0.09	1.40	0.87–2.26
Obesity	110	7.8	19	5.9	91	8.5	0.13	1.48	0.91–2.40
Epilepsy	57	4.1	6	1.9	51	4.8	0.02	2.06	0.88–4.82
Non-AIDS-related malignancies	49	3.5	9	2.8	40	3.7	0.42	1.25	0.61–2.59
Lung disease	37	2.6	9	2.8	28	2.6	0.87	0.91	0.43–1.90
Multimorbidity	852	61.1	153	47.4	699	65.3	< 0.0001	1.36	1.21–1.54

*ART* antiretroviral treatment, *CI* confidence interval, *CNS* central nervous system, *CVD* cardiovascular disease, *aRR* adjusted rate ratio

Sex, age class (50–59, ≥ 60 years), and HCV coinfection (except for liver disease) were included in the multivariate model.

**Fig. 1:**
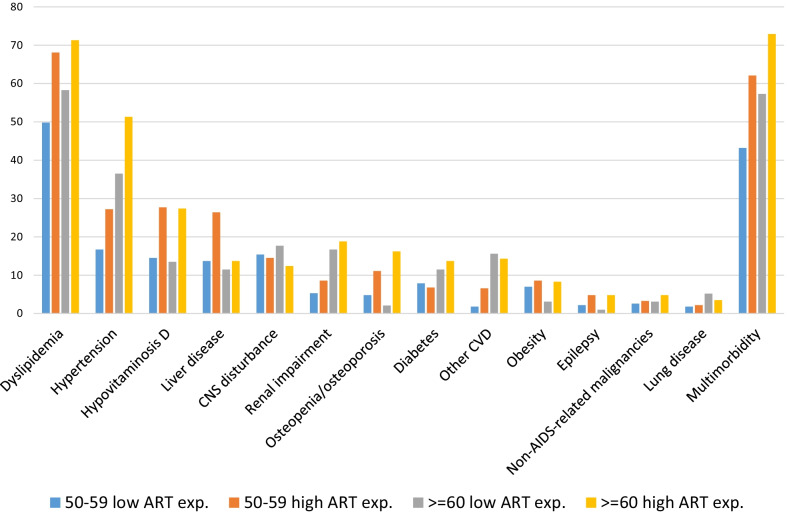
Prevalence of morbidities and multimorbidity, by age class and antiretroviral therapy (ART) exposure

Prevalence of comorbidities by age class (50–59 and ≥ 60 years) are shown in Fig. 1.

In the multivariate model, the findings were similar to those from the overall analysis, with prevalence of dyslipidemia, hypertension, hypovitaminosis D, osteopenia/osteoporosis and multimorbidity significantly higher in the ART exposed subjects (Table 3). However, we noted that higher exposure to ART was associated with CVD in PLWH aged 50–59, but not in those aged ≥ 60.

**Table 3:** Comorbidities by ART exposure, in strata of age

Comorbidity	Prevalence by ART exposure in strata of age							
	50–59				≥ 60			
	≤ 3 years	> 3 years	aRR	95% CI	≤ 3 years	> 3 years	aRR	95% CI
	N = 227	N = 757			N = 96	N = 314		
Dyslipidemia	49.8	68.1	1.40	1.22–1.62	58.3	71.3	1.26	1.05–1.52
Hypertension	16.7	27.2	1.61	1.16–2.23	36.5	51.3	1.48	1.10–1.99
Hypovitaminosis D	14.5	27.7	1.77	1.25–2.50	13.5	27.4	2.14	1.26–3.64
Liver disease	13.7	26.4	1.97	1.39–2.79	11.5	13.7	1.20	0.65–2.23
CNS disturbance	15.4	14.5	0.84	0.58–1.21	17.7	12.4	0.64	0.37–1.11
Renal impairment	5.3	8.6	1.48	0.81–2.73	16.7	18.8	1.30	0.74–2.28
Osteopenia/osteoporosis	4.8	11.1	2.02	1.09–3.74	2.1	16.2	7.54	1.87–30.37
Diabetes	7.9	6.8	0.91	0.52–1.58	11.5	13.7	1.28	0.69–2.38
Other CVD	1.8	6.6	2.93	1.06–8.13	15.6	14.3	1.02	0.60–1.73
Obesity	7.0	8.6	1.25	0.73–2.14	3.1	8.3	2.72	0.84–8.79
Epilepsy	2.2	4.8	1.68	0.46–4.30	1.0	4.8	4.24	0.56–32.00
Non-AIDS-related malignancies	2.6	3.3	1.17	0.48–2.89	3.1	4.8	1.42	0.41–4.88
Lung disease	1.8	2.2	0.71	0.24–2.10	5.2	3.5	0.68	0.24–1.94
Multimorbidity	43.2	62.1	1.41	1.19–1.65	57.3	72.9	1.30	1.08–1.57

*ART* antiretroviral treatment, *CI* confidence interval, *CNS* central nervous system, *CVD* cardiovascular disease, *aRR* adjusted rate ratio

Sex and HCV coinfection (except for liver disease) were included in the multivariate model.

In 2019, we started to collect information on CD4 + nadir. This data was available for about one-third of patients, equally distributed between groups on ART treatment duration. Including this variable in the model, the RRs were similar to those calculated without it (Tables 2, 3), although the confidence intervals were wider because of the lower sample size.

## Discussion

In this analysis of baseline data from the SCOLTA cohort study, we found that ART exposure is significantly associated with dyslipidemia, hypertension, and osteopenia/osteoporosis, hence with the presence of multimorbidity. On the contrary, diabetes, CVD, renal impairment, CNS disorders and non-AIDS-related malignancies were not associated with longer ART exposure. These results were confirmed in strata of age.

In the 50–59 group, longer ART duration was associated with increased risk of CVD, dyslipidemia and osteopenia/osteoporosis and, in general, of multimorbidity, but not of CNS disturbance and malignancies.

The higher number of over-sixties with dyslipidemia, hypertension, hypovitaminosis D and osteopenia/osteoporosis could be the effect of the exposure to more toxic antiretrovirals, like abacavir, protease inhibitors and tenofovir disoproxil fumarate. The relation of multimorbidity in geriatric HIV positive patients with a longer duration of HIV-infection and/or ART exposure can at least partially be attributed to the metabolic toxicities of ART, according to Guaraldi et al. [12]

On the contrary, the prevalence of CVD in older patients was similar among those with lower ART exposure, which presumably means a more recent diagnosis, maybe reflecting the fact that people with long-term exposure both to HIV and ART died prematurely.

In a recent issue of the FUNCFRAIL study [13], 801 PLWH were stratified by chronological age (50–54, 55–64 years, and > 65) and by the year of HIV diagnosis (before 1996 and after 1996). Among patients diagnosed before 1996, the authors found an increased burden of comorbidities and a higher prevalence of chronic obstructive pulmonary disease (COPD), history of cancer, osteoarthritis, depression, and other psychiatric disorders, but no differences regarding frailty and physical function; the prevalence of frailty and poor physical function was significantly higher among patients aged 65 years or more. Among patients aged ≥ 65 years, the most prevalent comorbidities were hypertension, diabetes, dyslipidemia, current cancer, and osteoarthritis. Despite the relevant difference between the results of the two studies, in the SCOLTA cohorts the two profiles of PLWH significantly differed in terms of comorbidities, as also was observed in the FUNCFRAIL study.

As regards the frequency of two or more concurrent medical conditions and their clusterization, data are scarce and difficult to compare, at least partly because of heterogeneous definitions of multi-morbidity [14, 15]. However, our findings are largely consistent with the results of other studies [10, 16, 17].

In Italy, comorbidity prevalence has been evaluated by Guaraldi et al. [16] in a sample of 2854 PLWH: using similar diagnostic criteria, previous CVD event and renal impairment prevalence estimates were largely similar, but we found a slightly lower proportion of subjects with diabetes (8.9% versus 11.1%) and a much higher proportion of hypertensive patients (31.6% versus 22.2%). In both studies age was the main determinant of multimorbidity, although in our sample male sex did not emerge as a risk factor for multimorbidity. However, in our sample the role of sex on different comorbidities was significant but not consistent throughout types: for example, women were at lower risk of liver disease and diabetes, but at higher risk for CNS disturbance and osteoporosis, thus resulting in an overall indifferent risk of multimorbidity.

In a previous study of our group [10], we found that multimorbid patients were about 54% and duration of ART was linked to higher probability of having 2 or more co-morbidities. The lower proportion of multimorbidity was likely due to the younger age of enrolled subjects.

In a cross-sectional study comparing 208 HIV positive subjects with 208 matched HIV negative controls [17], the frequency of multi-morbidity was higher in the former (63% vs 43%) than in the latter, and associated with duration of HIV and ART. In our sample, multimorbid patients were in a similar proportion, as expected since the age selection was similar.

Another interesting aspect was a comparison with the general Italian population, where the information was available [18]. An overall comparison was not feasible, since the age distribution of our patients was remarkably different. However, where information was provided in age class, we could compare some specific diseases (Additional file 1: Table S1).

For example, in a survey conducted in 2010, an overall 1.7% of men and 12.0% of women were reported as suffering from osteoporosis. Limiting the information to age classes comparable with ours, men aged 54–59, 60–64 and 65–74 years had a prevalence of osteoporosis of 2.2%, 1.7%, and 4.5%, respectively, versus 7.6%, 9.4% and 12.8% in our sample. On the contrary, in the same age classes the presence of osteoporosis in women was 18.0%, 21.2%, and 31.9% in the general Italian population, and 17.0%, 18.0% and 30.6% among women living with HIV. Similarly, CNS disturbance and hypertension were more frequent both in men and women from SCOLTA study than in the general population. Diabetes prevalence was comparable in both sexes.

In brief, comparing our data with those obtained from the general population, we can observe that, in the classes of age considered, over-fifties males are more affected by osteopenia/osteoporosis and, if over-sixties, also by high blood pressure and CNS disturbances. On the other hand, women living with HIV were more similar to the general population; this could be due to the success, in our outpatient facilities, of early diagnosis and treatment policy in post-menopausal HIV-positive women, a category considered at higher risk for osteopenia/osteoporosis.

This study has some limitations. First, the Infectious Diseases Clinics involved in the SCOLTA study are not formally representative of the Italian Clinics (i.e., at the national level), because they were not randomly selected but participated in our observational study on a volunteer basis. Second, the subjects were not fully representative of all PLWH followed in the Infectious Diseases Clinics participating into the SCOLTA study, but only of those in need of initiating a new ART drug in the considered period. Further, although we chose HCV coinfection over history of IDU to be included in the adjusted model, we cannot exclude an additional effect of IDU on comorbidity, because of the almost complete overlap of IDU and HCV coinfection. Moreover, since we did not collect the year of HIV infection diagnosis, we could not evaluate the role of duration of exposure to HIV as a risk factor. Another factor not evaluable was the association between exposure to specific drugs and comorbidities: as a matter of fact, we collected the overall duration of NRTI, NNRTI, PI, and INSTI use in classes, that included drugs with very different toxicities. A further point was that no well-defined cutoff is currently defined to identify a low exposure to ART. Considering that the majority of the non-communicable diseases observed among PLWH (especially metabolic and cardiovascular) needs a few years of exposure to risk factors to exert their effect, we chose a 3-year period as a reasonable cutoff. Then again, the sample size was limited, in particular as regards comorbidities with low prevalence, such as epilepsy, non-AIDS-related malignancies, and pulmonary diseases. From a statistical point of view, our analyses were not adjusted for multiple comparisons, thus some of the nominally significant RRs might be false positives, although a rationale existed for our findings. Last, comorbidities were only reported if previously diagnosed, so an underestimate was possible.

In conclusion, HIV outpatient facilities nowadays provide, in our country, an opportunity for a 360 degrees assessment of comorbidities. Consequently, PLWH adherent to their schedule could be better followed for non-communicable diseases with respect to the general population. This represents a real achievement of the last decades for these patients that should be preserved, even though, unfortunately, the Covid19 pandemic and the post-Covid19 era could seriously jeopardize this advantage. In the screening of non-communicable diseases, PLWH over-fifties present different risk profiles for comorbidities, according to their ART exposure, and should not be considered as a uniform group. Distinguishing them according to different profiles may contribute to the design of different diagnostic and therapeutic approaches.

## Supplementary Information

**Additional file 1: Table S1.** Comorbidities by age class (%), comparison with the general Italian population.

## Data Availability

The datasets analyzed during the current study are not publicly available due to restrictions imposed by the Ethics Committees but are available from the corresponding author on reasonable request.

## Abbreviations

AIDS: Acquired immunodeficiency syndrome
aRR: Adjusted rate ratio
ART: Antiretroviral therapy
BMI: Body mass index
CDC: Center for Diseases Control
CI: Confidence interval
CISAI: Coordinamento Italiano per lo Studio di Allergia e Infezioni da HIV
CVD: Cardiovascular diseases
eGFR: Estimated glomerular filtration rate
HCV Ab+: Antibodies for hepatitis C virus
HIV: Human Immunodeficiency Virus
INSTI: Integrase strand transfer inhibitors
IDU: Intravenous drug use
IQR: Interquartile range
NNRTI: Non nucleoside reverse transcriptase inhibitors
RR: Rate ratio
PI: Protease inhibitors
PLWH: Persons living with HIV
SD: Standard deviation
