# Supplementary material for: Growing old with antiretroviral therapy or elderly people in antiretroviral therapy: two different profiles of comorbidity?
Source: BMC Infect Dis. 2022 Sep 23;22:745. doi: 10.1186/s12879-022-07739-y (PMC9508769; doi:10.1186/s12879-022-07739-y)
Supplement: Supplementary file 1 — Additional file 1: Table S1. Comorbidities by age class (%), comparison with the general Italian population. [file 12879_2022_7739_MOESM1_ESM.docx]

**Supplemental Table.** Comorbidities by age class (%), comparison with the general Italian population.

|  | **M** | | | **F** | | |
| --- | --- | --- | --- | --- | --- | --- |
|  | **55-59**  **N=328** | **60-64**  **N=171** | **65-74**  **N=133** | **55-59**  **N=94** | **60-64**  **N=39** | **65-74**  **N=36** |
| Diabetes  ISTAT  SCOLTA | 7.4  9.4 | 9.4  11.7 | 12.6  13.5 | 5.9  5.2 | 7.3  5.1 | 13.1  13.9 |
| Hypertension  ISTAT  SCOLTA | - | - | 43.4  57.1 | - | - | 48.3  55.6 |
| CNS disturbance  ISTAT  SCOLTA | - | - | 7.3  12.8 | - | - | 11.6  25.0 |
| Osteoporosis  ISTAT  SCOLTA | 2.2  7.6 | 1.6  9.4 | 4.5  12.8 | 18.0  17.0 | 21.2  18.0 | 31.9  30.6 |
